# Cryopreservation of human failed-matured oocytes followed by in vitro maturation: vitrification is superior to the slow freezing method

**DOI:** 10.1186/1477-7827-9-156

**Published:** 2011-12-12

**Authors:** ZhiGuo Zhang, Yu Liu, Qiong Xing, Ping Zhou, Yunxia Cao

**Affiliations:** 1 Reproductive Medicine Centre, Department of Obstetrics and Gynecology, First Affiliated Hospital of Anhui Medical University, The People's Republic of China; 2 Anhui Provincial Key Laboratory of Population Health and Aristogenics, the People's Republic of China

**Keywords:** cryopreservation of human failed-matured oocytes, slow-freezing, vitrification, survival rate, *in vitro *maturation rate, fertilization, development rates

## Abstract

**Background:**

Oocyte cryopreservation is an important method used in a number of human fertility circumstances. Here, we compared the survival, *in vitro *maturation, fertilization, and early embryonic development rates of frozen-thawed human immature oocytes using two different cryopreservation methods.

**Methods:**

A total of 454 failed-matured oocytes [germinal vesicle (GV) and metaphase I (MI) stages] were collected from 135 patients (mean age 33.84 +/- 5.0 y) who underwent intracytoplasmic sperm injection (ICSI) cycles between February 2009 and December 2009 and randomly divided into a slow freezing group [1.5 mol/L-1, 2-propanediol (PROH) + 0.2 mol/l sucrose] and vitrification group [20% PROH + 20% ethylene glycol (EG) + 0.5 mol/l sucrose].

**Results:**

The vitrification protocol yielded a better survival rate than the slow freezing protocol at each maturation stage assessed. Regardless of the maturation stage (GV + MI), the slow freezing protocol had a significantly lower survival rate than the vitrification protocol (p < 0.001). In addition, a significant difference was found in the survival rates between GV and MI oocytes regardless of the protocol used (90.1 vs. 64.7%, respectively; p < 0.01). We also found that the maturation rates of GV and MI oocytes from the slow freezing and vitrification groups were 16.7 vs. 24.4% and 50.8 vs. 55.4%, respectively. Regardless of the protocol used, the GV oocytes had significantly lower viability than MI oocytes after 36 h of *in vitro *maturation (21.2 vs. 54.0%, respectively; p < 0.01). In addition, the GV and MI oocytes from the slow freezing group had a markedly lower maturation rate than those from the vitrification group (33.6 vs. 43.1%, respectively), but no statistical difference was found between the two groups (P > 0.05). For the GV-matured oocytes, no fertilized eggs were obtained in the slow-freezing group, while a 19.0% (4/21) fertilization rate was observed in the vitrification group. For the MI-matured oocytes, fertilization rates for the slow freezing and vitrified groups were 36% and 61.1%, respectively, but no significant difference was found between the two groups (PIn the Methods section in the MS, all procedures were compliant with ethical guidelines, i.e. approved by the Ethical Committee of our university and Informed Consent signed by each patient. > 0.05). In the GV vitrification group, no embryo formed; however, in the MI slow freezing group, 12 oocytes were fertilized, but only two achieved cleavage and were subsequently blocked at the 2-cell stage. In the MI vitrification group, a total of 22 embryos were obtained, five of which developed to the blastocyst stage.

**Conclusions:**

Vitrification is superior to the slow freezing method in terms of the survival and developmental rates for the cryopreservation of human failed-matured oocytes. In addition, GV oocytes appeared to be more resistant than MI oocytes to the low temperature and cryoprotectant used during cryopreservation.

## Background

Oocyte cryopreservation is an important method used to preserve female fertility under different pathological conditions [1] and avoids ethical issues that are associated with embryo cryopreservation. This method provides the safe storage of excess oocytes during assisted reproductive technology (ART) therapies and improves the establishment of oocyte banks. Oocyte preservation can also provide a solution for cancer patients wishing to preserve fertility before undergoing chemotherapy or radiation therapy, or for young cancer patients who do not as yet have a male partner [2]. When the risk and delay of stimulation are unacceptable for patients, the cryopreservation of immature oocytes followed by *in vitro *maturation (IVM) can solve this problem [3]. The main problem associated with cryopreservation of metaphase II oocytes (mature oocytes) is the sensitivity of microtubule spindles to low temperatures and cryoprotectants [4]; however, this can be avoided by cryopreserving immature oocytes, which have chromosomes within the nuclear membrane that are not yet arranged on the microtubule spindles [5]. The location of the chromosomes in immature oocytes is thought to provide protection from the direct exposure to low temperature and cryoprotectants during freezing and thawing [5]. Cryopreservation of immature oocytes is a method for circumventing spindle damage, but the main disadvantage of cryopreservation of immature oocytes is the fact that IVM is required after thawing. Although IVM is routine in some animal species, it is not easily performed with human oocytes, and very few successful pregnancies from cryopreserved immature oocytes have been reported [6,7].

There are two major techniques for oocyte cryopreservation: slow freezing and vitrification. To date, no universal slow freezing protocol exists. Computer-controlled automatic freezing devices are often used for slow freezing. In general, the standard slow freezing protocol consists of two stages: freezing and thawing. The vitrification process brings the samples to an extremely low temperature in a glassy state in order to avoid the formation of ice crystals. This is achieved by combining the use of a high concentration of cryoprotective solution with an extremely rapid cooling speed. Vitrification has the advantage of being carried out rapidly and easily and does not require expensive equipment. However, it can also easily expose samples to osmotic shock and toxicity due to the high concentration of cryoprotectant used, which may affect the viability of the embryo/oocyte. The common vitrification protocol for embryo/oocyte cryopreservation also has two stages: cooling and warming. The majority of studies have shown that vitrification methods are more efficient and reliable than any version of the slow freezing method [8-10].

In the present study, the cryopreservation of failed-matured oocytes collected from intracytoplasmic sperm injection (ICSI) therapy cycles was performed in order to explore cryopreservation of human immature oocytes. In addition, we aimed to develop a new option for the cryopreservation of human oocytes and provide a better method for preserving fertility.

## Methods

### Study period and patients

A total of 454 failed-matured oocytes obtained from 135 patients (mean age 33.56 +/- 4.0 y) who underwent ICSI cycles between February 2009 and December 2009 were used for this study. ICSI treatment was performed due to male infertility, oligozoospermia, and obstructive azoospermia. The study was initially approved by the ethical and scientific committee of the first affiliated hospital of Anhui Medical University, China. Patients included in the study were thoroughly informed and signed written consent forms. All procedures in the Methods section were compliant with ethical guidelines, i.e. approved by the Ethical Committee and Informed Consent signed by each patient.

### Stimulation protocol and oocyte recovery procedures

The patients were given standard ovarian stimulation using a long or short protocol. After down-regulation with a gonadotropin-releasing hormone antagonist (Dipherline, IPSEN, France), the patients were stimulated with human menopausal gonadotropin (r-FSH, Serono, Switzerland; HMG, Lizhu, China). The oocyte retrieval was performed through vaginal puncture under ultrasound guidance. After ovum pick-up, oocytes were enzymatically denuded of cumulus cells with 60-80 mU/ml Hyaluronidase solution (Sigma, USA) in order to assess nuclear maturity. All retrieved metaphase II (MII) oocytes were used for patients' treatments, and the germinal vesicle (GV) and metaphase I (MI) oocytes, which are also called failed-matured oocytes, were divided into either the slow freezing or vitrification groups through a random computer-generated list. Prior to cryopreservation, the oocytes were evaluated microscopically based on morphology to identify high-quality oocytes with an appropriate size, normal zona pellucida, and integral membrane.

### Protocols for oocytes slow freezing

To freeze, the failed-matured oocytes were first placed in equilibration solution, which was composed of HTF1023 (SAGE, USA), 15% serum protein substitute (SPS, SAGE, USA), and 1.5 mol/L-1.2-propanediol (PROH, Sigma, USA) for 10 min at room temperature (RT), and then placed in freezing solution at RT for 1 min, which was composed of HTF1023 with 15% SPS, 1.5 mol/L-PROH, and 0.2 mol/l sucrose. Subsequently, a small amount of the freezing solution containing oocytes was loaded into 0.25 ml plastic straws (MINITüB, Japan). The plastic straws were then transferred into an automated freezing machine (CL-8800, Australia) with an initial chamber temperature of 21°C. The temperature was reduced to -7°C at a rate of -2°C/min and ice seeding was induced manually. After a holding time of 10 min at -7.0°C, the straw was slowly cooled to -35°C at a rate of -0.3°C/min and then rapidly cooled to -80°C at a rate of -45°C/min. After 15 min at the stabilization temperature, the straws were immediately placed into liquid nitrogen for a minimum of one month of storage.

To thaw the samples, the plastic straws were first air-warmed for 40 s and then immersed in a 31°C water bath for 60 s for complete removal of all ice.The cryoprotectants were removed at RT in the following solutions: HTF1023 with 15% SPS and 0.5 mol/l sucrose (37°C, 10 min), HTF1023 with 15% SPS and 0.3 mol/l sucrose (37°C, 10 min), and HTF1023 with 15% SPS (37°C, 7 min).

### Protocol for oocytes vitrification

The oocytes were vitrified in equilibration medium [HTF1023, 30% SPS, 7.5% (v/v) PROH, and 7.5% (v/v) EG] at RT for 5-9 min, followed by incubation in vitrification medium [HTF1023, 30% SPS, 0.5 M sucrose, 15% (v/v) PROH, and 15% (v/v) EG] at RT for 30 s. The samples were then loaded into cryotops (KITAZATO Biopharma, Japan) at a volume less than 1 μl and immediately submerged into liquid nitrogen for at least 1 month of storage.

To thaw the vitrified oocytes, the cryotops were taken out of the liquid nitrogen and gradually warmed to RT in the following solutions: Thawing Medium: HTF1023 + 30% SPS + 1.0 M Sucrose (37°C, 1 min); Diluent Medium I: HTF1023 + 30% SPS + 0.5 M Sucrose (37°C, 3 min); Diluent Medium II: HTF1023 +30% SPS + 0.25 M Sucrose (37°C, 3 min); Washing Medium I: HTF1023 + 30% SPS (37°C, 3 min); and Washing Medium II: HTF1023 + 30% SPS (37°C, 3 min)

After thawing, all of the oocytes were cultured in fertilization medium (Cook, USA) at 37°C, 6% CO_2_, and 5% O_2 _and checked after 1 h for survival based on the membrane integrity. If the rim of the cytoplasmic periphery appeared as a smooth arc under an inverted microscope, it indicated that the oocyte was viable; if the rim was irregular and the cytoplasm was diffuse, then the oocyte was not viable.

### Protocol for IVM, ICSI, and embryo culture

The frozen-thawed immature oocytes were cultured in self-prepared IVM medium supplemented with 75 mUI/ml of FSH (Gonal-F, Serono, Swiss), 75 mIU/mlhCG (Profasi, Serono, Swiss) and 20% patient serum [11] in a humidified atmosphere at 6% CO_2 _and 5% O_2 _at 37°C. The appearance of the first polar body (PB1) indicated oocyte maturation during culture. The GV and MI oocytes were cultured for 24 h and the mature oocytes with PB1 were isolated and inseminated by ICSI using sperm donors who had signed informed consent forms. The remaining oocytes were placed back into culture for up to an additional 12 h, and oocyte maturation was observed every hour during the incubation period.

The *in vitro *matured oocytes were inseminated by ICSI using sperm donors. Injected oocytes were individually cultured in micro-drops containing cleavage medium (Cook, USA) at 37°C, 6% CO_2_, and 5% O_2_. Fertilization was assessed 16-18 h after the injection for the appearance of two pronuclei and two polar bodies. The formed embryos were cultured for 2-3 d in the cleavage medium and the embryonic development was evaluated daily by scoring fragmentation as well as the number and appearance of blastomers.

Data were compared using χ^2 ^tests. Yates corrected p values less than 0.05 were regarded as statistically significant.

## Results

### Freezing and thawing: survival rates

A total of 454 failed-matured oocytes were randomized into two different groups. The survival rates of oocytes from the two freezing groups at different developmental stages are shown in Table 1. Regardless of the maturation stage (VG + MI), the slow freezing method induced a significantly lower survival rate of 62% (119/192) compared to 82% (216/262) for the vitrification method (p < 0.01). Survival rates of the GV oocytes were 85.7% (60/70) for the slow-freezing and 93.5% (86/92) for the vitrification methods, and survival rates of the MI oocytes were 48.4% (59/122) and 76.5% (130/170) for the slow freezing and vitrification methods, respectively. Regardless of the protocol used, a significant difference was found in the viability between the GV and MI oocytes (90.1 vs. 64.7%, respectively; p < 0.01). These results indicated that GV oocytes were more resistant to the freeze/thaw procedure than the MI oocytes. In addition, the vitrification protocol provided a better survival rate than the slow-freezing protocol.

**Table 1:** Comparison of the survival rates between the slow-freezing and vitrification groups

	Slow-freezing	Vitrification	Total
GV + MI collected	192	262	454
GV collected	70	92	162
MI collected	122	170	292
GV + MI survival (%)	119 (62.0)	216 (82.4) *	335 (73.8)
GV survival (%)	60 (85.7)	86 (93.5)	146 (90.1)
MI survival (%)	59 (48.4)	130 (76.5) *	189 (64.7) **

*Significantly different between the slow-freezing and vitrification groups (P < 0.01)

**Significantly different between the number of GV and MI that survived (P < 0.01)

### In vitro maturation rates

As shown in Table 2, the maturation rate of the GV oocytes was 16.7% (10/60) under the slow freezing process and 24.4% (21/86) under the vitrification process. In addition, the maturation rates for the MI oocytes were 50.8% (30/59) for the slow freezing process and 55.4% (72/130) for the vitrification process. Regardless of the protocol used, the GV oocytes had significantly worse viability after 24-36 h of IVM than the MI oocytes (21.2 vs. 54.0%, respectively; p < 0.01). Regardless of the maturation stage, the oocytes from the slow freezing process had a markedly lower maturation rate than those from the vitrification process (33.6 vs. 43.1%, respectively); however, no statistical difference was found between the two groups (p > 0.05).

**Table 2:** Status on maturation, fertilization and development of failed-matured oocytes that survived cryopreservation

	Viable GV Oocytes		Viable MI Oocytes	
	Slow-freezing	Vitrification	Slow-freezing	Vitrification
No. of oocytes matured *in vitro *(%)	31 (21.2) total		102 (54.0)* total	
	10 (16.7)	21 (24.4)	30 (50.8)	72 (55.4)
No. of fertilized (%)	4 (12.9) total		60 (58.8)* total	
	0 (0.0)	4 (19.0)	12 (36.0)	44 (61.1)
No. of cleaved (%)	0 (0.0) total		24 (40.0) total	
	0 (0.0)	0 (0.0)	2 (16.7)	22 (50.0)**
No. of blastocysts formed (%)	0 (0.0) total		5 (20.8) total	
	0 (0.0)	0 (0.0)	0 (0.0)	5 (22.7)

*Significantly different between the GV and MI oocytes that survived (P < 0.01)

** Significantly different between the slow-freezing and vitrification groups (*P *< 0.05)

### Fertilization and development

All of the *in vitro *matured oocytes were inseminated by ICSI using donated semen and fertilization was assessed 16-18 h after the injection for the appearance of two pronuclear and two polar bodies (Table 2). For the GV-matured oocytes, no fertilized eggs formed from the slow freezing group, while a 19.0% (4/21) fertilization rate was achieved in the vitrification group. For the MI-matured oocytes, fertilization rates were 36.0% (12/30) and 61.1% (44/72) for the slow freezing and vitrification groups, respectively, without a significant difference between the two groups (p > 0.05). Regardless of the protocol used, the MI oocytes had a significantly higher fertilization rate than the GV oocytes (58.8 vs. 12.9%, respectively; p < 0.01) following cryopreservation, IVM, and insemination.

In the GV vitrification group, no embryo formed. In addition, of the 12 fertilized oocytes in the slow freezing MI group, only two oocytes achieved cleavage, which were subsequently blocked at the 2-cell stage. In the MI vitrification group, a total of 22 embryos formed, and of them, five embryos developed to the blastocyst stage. Importantly, there was a significant difference in the cleavage rates between the two groups (50.0 vs. 16.7%, respectively; p < 0.05).

## Discussion

In routine IVF or ICSI cycles, some immature oocytes, called failed-matured oocytes, are often observed during oocyte retrieval. These oocytes are generally considered unusable since they have a low potential to develop into an embryo. In 2004, Chian *et al. *[12] reported pregnancies and live births as a result of IVF with oocytes retrieved during the natural cycle and subjected to IVM. Based on these important findings, we hypothesized that some of the failed-matured oocytes may be competent for the full-term development of a fetus after IVM, fertilization, and *in vitro *culture. Therefore, these failed-matured oocytes may provide a benefit for patients, oocyte donation, and the study of the cryopreservation of human immature oocytes.

The cryopreservation of human immature oocytes has distinct advantages compared to mature oocytes. Immature oocytes may circumvent the problem of spindle damage that frequently occurs in mature oocytes during cryopreservation, since the chromosomes remain within the nucleus [5]. This unique position protects them from direct exposure to low temperatures and cryoprotectants [4]. Moreover, the cryopreservation of immature oocytes could allow cancer patients to preserve fertility before receiving chemotherapy or radiation therapy without delaying treatment or experiencing other risks due to oocytes induction [3]. However, the cryopreservation of human immature oocytes also has disadvantages, and frozen and thawed immature oocytes from IVM followed by insemination have been shown to have lower fertilization and cleavage rates than mature oocytes. To date, very few human live births have been reported following the cryopreservation of GV stage oocytes and IVM [13-15]. Therefore, the cryopreservation of mature oocytes is still a more efficient method than the cryopreservation of immature oocytes [16-18]. For substantial progress to be made in the cryopreservation of human immature oocytes, more effort and creativity should be placed on studies that explore these methods.

In the process of performing ICSI, failed-matured oocytes are often observed and easily collected. In addition, some failed-mailed oocytes have been reported to have the potential to develop healthy offspring [12]. Based on these findings, we used failed-matured oocytes to study cryopreservation methods of human immture oocytes. The extent of cell injury in the failed-matured oocytes at each different stage was evaluated in detail following the freeze/thaw process after slow freezing or vitrification protocols were performed.

In this study, we found a significant difference in the viability of oocytes between the vitrification and slow freezing protocols regardless of the maturation stage. These findings support the hypothesis that vitrification provides better viability of failed-matured oocytes than the slow freezing technique. Importantly, five blastocysts were obtained from the vitrification group, whereas no blastocyst formed from the slow-freezing group. Although the IVM rate in our study was not significantly different between the two groups, the vitrification procedure still achieved better results. Therefore, these results demonstrate that the vitrification process is superior to the slow freezing method used for cryopreservation of human failed-matured oocytes. According to our fertilization and developmental results, we hypothesize that vitrification provides better preservation of microtubule organization and a reduction of cytoskeletal spindle damage compared to the slow freezing method.

In the present study, we found that GV oocytes had a significantly higher viability than MI oocytes regardless of the protocol used. These results suggest that GV oocytes are more resistant to the freeze/thaw procedure than MI oocytes, which may be related to the unique cellular structure present at the GV stage. GV oocytes have large germinal vesicles and two large cell membranes: a cytoplasmic membrane and GV membrane. During the freeze/thaw process, the two membranes of GV oocytes may be more resistant to osmotic pressure changes and therefore avoid the disintegration of the cytoplasmic membrane, which can occur in MI oocytes.

In conclusion, the study presented here has demonstrated that vitrification is superior to the slow freezing method in terms of survival and developmental rates of human failed-matured oocytes that have been cryopreserved. In addition, GV oocytes appeared to be more resistant to the low temperature and cryoprotectant than the MI oocytes during the cryopreservation process. Since discarded oocytes were used for this study, additional studies are needed with a larger sample number in order to definitively explore the use of human failed-matured oocytes in cryopreservation for human fertility.

## Competing interests

The authors declare that they have no competing interests.

## Authors' contributions

ZZ, YL, and QX collected all samples and performed the study. ZZ drafted the manuscript. PZ and YC participated in the design and coordination and helped to review the manuscript. All authors read and approved the final manuscript.
